# Did Dog Domestication Contribute to Language Evolution?

**DOI:** 10.3389/fpsyg.2021.695116

**Published:** 2021-09-13

**Authors:** Antonio Benítez-Burraco, Daniela Pörtl, Christoph Jung

**Affiliations:** ^1^Department of Spanish, Linguistics, and Theory of Literature (Linguistics), Faculty of Philology, University of Seville, Seville, Spain; ^2^Psychiatric Department, Saale-Unstrut Klinikum, Teaching Hospital Leipzig and Jena Universities, Naumburg, Germany; ^3^Petwatch, Halle, Germany

**Keywords:** dog domestication, human self-domestication, aggression, prosociality, language evolution, cognitive disorders

## Abstract

Different factors seemingly account for the emergence of present-day languages in our species. Human self-domestication has been recently invoked as one important force favoring language complexity mostly via a cultural mechanism. Because our self-domestication ultimately resulted from selection for less aggressive behavior and increased prosocial behavior, any evolutionary or cultural change impacting on aggression levels is expected to have fostered this process. Here, we hypothesize about a parallel domestication of humans and dogs, and more specifically, about a positive effect of our interaction with dogs on human self-domestication, and ultimately, on aspects of language evolution, through the mechanisms involved in the control of aggression. We review evidence of diverse sort (ethological mostly, but also archeological, genetic, and physiological) supporting such an effect and propose some ways of testing our hypothesis.

## Introduction

Over the last decades, language evolution has emerged as a favorite topic of inquiry for researchers from different fields, from linguists to anthropologists to ethologists to prehistorians. Language is a hallmark of the human distinctive phenotype, enabling sophisticated ways of thinking, communicating information, and organizing human societies. For many years, a sharp divide was established between the evolution of the cognitive and behavioral hardware that enables us to learn and use languages (aka *faculty of language*, *language-ready-brain*, or more recently, *human linguisticality*) and the particular codes we use in our daily interactions (aka *languages*). Whereas the former was hypothesized to result from biological changes mostly (aka *language evolution*), present-day languages were hypothesized to derive from the first language(s) used by first anatomically modern humans (AMHs) via random modifications triggered by external factors, like social and geographical isolation or cultural exchanges (aka *language change*). Putting this differently, although many languages are currently spoken by human beings, they have been thought to exhibit similar core properties because these properties are imposed by our brain architecture. Chomskyan (evolutionary) linguistics is a distinct example, with its claim that language as a cognitive faculty emerged quite recently and has not undergone modification since then, whereas individual languages have changed over time, but with this change being constrained by this basic, uniform, and unaltered cognitive and behavioral framework (Bolhuis et al., 2014).

Increasing evidence (reviewed e.g., by Benítez-Burraco, 2019) suggests instead that our cognitive and behavioral hardware might have gone on evolving after the emergence of AMHs. For instance, our characteristic globular skull/brain, which is hypothesized to account for some of our distinctive cognitive abilities (Boeckx and Benítez-Burraco, 2014), can only be attested in recent specimens of AMHs (Neubauer et al., 2018). Likewise, several genes involved in brain development and function show signals of positive selection well after our split from extinct hominins (Zhou et al., 2015). Our behavior has equally changed with time, with evidence of modern human behavior and culture being widespread only from 100 to 50 thousand years ago (kya) onward (McBrearty and Brooks, 2000; Mellars, 2002; Henshilwood and Marean, 2003). At the same time, languages have been shown to be constrained by (or even adapt to) environmental factors, mostly cultural, but physical too, with some core properties of human language (like duality of patterning) emerging via a cultural process as languages are learned and transmitted by speakers (Sandler et al., 2005; Lupyan and Dale, 2010). Ultimately, the different languages we learn and use have a differential impact on selected cognitive abilities, like working memory (e.g., Amici et al., 2019), and eventually on our cognitive architecture, particularly if cognitive gadgets aimed to process linguistic information more quickly and efficiently are implemented (*cognitive gadgets* are conceived by Heyes, 2018 as cognitive tools or mechanisms that are not genetically encoded, but that are culturally developed and transmitted through social interaction). All this evidence suggests that, better than the outcome of a linear evolutionary process (with *language evolution* occurring first and *language change* happening later), the emergence of modern languages should be viewed as an (ongoing) feedback loop between our biological endowment and our cultural practices, with language features resulting from and impacting on our faculty of language in a virtuous circle.

In biological sciences, *niche construction theory* has recently emerged as a robust theory aiming to account for these feedback loops between biology and culture. It can be thus expected to also provide a rationale for most aspects of human evolution. Under this view, our biology enables us to construct a cultural environment or niche (encompassing ways of life in a broad sense, from clothe making to food supplies strategies to transmission of know-hows) that modifies, reduces, or even eliminates many of the selective pressures most animals need to cope with and which they mostly address via biological changes. Certainly, many other animal species exhibit some form of culture that increases their survival rates in the absence of biological modifications, but in our species cultural niche construction is widespread and seemingly accounts for many crucial aspects of our distinctive phenotype (Laland et al., 2000; Kobayashi et al., 2019). Language is not an exception (Sinha, 2015).

The domestic environment, which also results in a relaxation of selective pressures, can be construed as a sort of niche. Interestingly, domestication has been claimed to trigger some common features in mammals: this is the so-called “domestication syndrome” (Wilkins et al., 2014). Although it is disputed that this syndrome is a universal outcome of all domestication processes, some common changes in behavior (like increased tameness) and in cognitive abilities (like enhanced social learning) via changes in the brain (like a reduction in brain size) are usually observed in most domesticated animals (see Sánchez-Villagra et al., 2016 for discussion). When compared to extant apes and extinct hominins, humans exhibit many of the traits found in domesticates (see Shea, 1989; Leach, 2003; Somel et al., 2009; Zollikofer and Ponce de León, 2010; Herrmann et al., 2011; Plavcan, 2012; Stringer, 2016; Hare, 2017 for different features), hence the claim that we were engaged in some sort of domestication process too. This is the hypothesis of *human self-domestication*. According to this view, our “domestication” (or more neutrally, the reduction in reactive aggression levels observed in our species) accounts for many aspects of our complex social and cultural practices, including our notable cooperation, our improved social cognition, the widespread social networks we form (also encompassing non-kin people), and our sophisticated technology (Hare, 2017). Because these effects are hypothesized to have resulted mostly from changes in our behavior, self-domestication can be viewed as a sort of niche construction due to cultural evolution processes within general evolution.

Interestingly enough, in songbird species, domestication increases song complexity (Takahasi and Okanoya, 2010; Kagawa et al., 2012; Okanoya, 2017). This is seemingly due to the changes in the glutamate-dopamine signaling in the striatum brought about by the attenuated stress signaling under domestication, which results in more variable vocalizations (O’Rourke et al., 2021). These findings paved the way toward claims that domestication might be involved in the evolution of human language too. Although a comprehensive model of language evolution under the effects of self-domestication is still pending, some hints can be found in the recent literature. For instance, Thomas and Kirby (2018) have argued that our self-domestication fueled the processes that enable the cultural evolution of language, particularly, the transmission of communicative systems through learning, as well as the ability to infer the communicative intent associated with a signal or action. Likewise, in a series of related papers, Progovac and Benítez-Burraco (2019), Benítez-Burraco et al. (2020), and Benítez-Burraco and Progovac (2020) have hypothesized in some more detail how changes in the management of aggression as a result of our increased self-domestication might have contributed to make language structure and use more complex via its impact on behaviors needed for acquiring and mastering a language (like language learning by children, language teaching by adults, or language play), with self-domestication and language complexity being engaged in a positive feedback loop. They have also advanced a potential neurobiological mechanism accounting for this effect (see Benítez-Burraco and Progovac, 2021 for details). In brief, the same neuronal mechanism, involving a widespread connectivity between cortical and subcortical structures, is responsible for the suppression/inhibition of reactive aggression and for syntactic chunking. Consequently, as self-domestication increased, language structures and uses also became more complex. At the same time, using more complex structures would have contributed to increasing the cortical control of subcortical mechanisms involved in aggression, particularly if these early forms of language were used for expressive purposes mostly (perhaps for verbal contests), thus replacing, at least partially, physical aggression, which is more harmful. Overall, this would have resulted in a further reduction of reactive aggression that fostered our prosociality further, as well as the other hypothesized consequences of self-domestication. In other words, these authors expect language and reactive aggression to be engaged in a mutually reinforcing feedback loop, resulting in increased language complexity and increased behavioral prosociality.

Hypotheses of this sort are difficult to test, as languages leave no trace in the fossil or archeological records. To test them, we need to rely on proxies or windows to previous stages in the evolution of language (and of languages), provided that the proper bridging inferences can be postulated. One useful proxy of this sort is animal models. Recent comparative approaches to the evolution of human language have concluded that its basic building blocks are shared with other animals (although some human-specific innovations can be expected too). Additionally, it seems that other animal species have gone through a process of self-domestication, particularly bonobos (Hare et al., 2012). Accordingly, the aim of this paper is twofold. On the one hand, we wish to argue that dog domestication can be regarded a useful model of the changes that happened in human cognition and behavior under the effect of self-domestication, with a focus on our communicative abilities, particularly language. Several lines of evidence support this approach. First, contrary to other species, dogs exhibit most of the distinctive features encompassing the “domestication syndrome” (Sánchez-Villagra et al., 2016). Second, as we discuss in detail in the next section, dog domestication resulted from selection for tameness, pretty much like human self-domestication did. Third, in dogs, domestication results in the enhancement of cognitive abilities and behaviors that are crucial for language acquisition and use, like the sensibility to social cues or the ability to solve problems relying on social cues (Hernádi et al., 2012; Udell, 2015). Finally, common genetic determinants might account for the enhanced sociability associated to dog domestication and to human self-domestication. Hence, genes selected in dogs overlap with genes selected in AMHs (Theofanopoulou et al., 2017). More importantly, some chromosomal regions known to be under positive selection in dogs are associated with aspects of our distinctive social behavior, like the region deleted in Williams syndrome (WS) (vonHoldt et al., 2017). The fact that people with WS exhibit more marked features of human self-domestication (Niego and Benítez-Burraco, 2019) makes this connection more intriguing.

Nonetheless, the paper also aims to explore the more debatable possibility that human self-domestication and dog domestication were engaged in some sort of positive feedback loop and that this mutual reinforcement contributed to language evolution in some subtle, but still important, way. In truth, this is not a totally new idea. In particular, Groves (1999, p. 111) has famously claimed that “The human-dog relationship amounts to a very long-lasting symbiosis […] and intensified in the Holocene into mutual domestication. Humans domesticated dogs and dogs domesticated humans.” That said, this possibility has not been properly tested, and it has certainly not been applied to the domain of language and communication. Again, several lines of evidence support this possibility. First, although genetic analyses have dated the origins of dog domestication to around 15 kya (Freedman et al., 2014; Freedman and Wayne, 2017), dog (proto)domestication seemingly started during the Upper Paleolithic, perhaps as early as 35 kya (Galibert et al., 2011), a period when features of human self-domestication reached its peak (Cieri et al., 2014). Second, closely living and working together with a different species has been shown to reduce stress and therefore the activity of hypothalamic-pituitary-adrenal (HPA) axis, this circumstance usually resulting in enhanced sociability, or even hypersociability, which is the physiological factor that ultimately promotes all the changes associated with (self-)domestication (Jung and Pörtl, 2015). Third, and related to this, dog domestication made human social networks larger and more complex by including members of other species; this circumstance seemingly demanded enhanced tolerance to non-kin as well as extra emotional bonding, and perhaps improved mindreading too. Contact with non-kin and, ultimately, the establishment of intergroup social networks (but also the management of intergroup conflicts) have been hypothesized to account for specific types of human languages (see Benítez-Burraco and Progovac, 2020 for details), as well as for the emergence of modern use of languages, that is, modern pragmatics (Benítez-Burraco et al., 2020).

The paper is structured as follows. We first provide a brief characterization of human self-domestication vis-à-vis dog domestication, highlighting the parallels between the two processes. We then explore the effects of human-dog interactions on their respective behavioral and cognitive phenotypes, with a focus on aspects known to be affected by (self-)domestication. Finally, we focus on communication, both verbal and non-verbal, and advance some conjectures about the putative effects of this coexistence (and possibly, co-evolution) on the emergence of present-day languages.

## Human Self-Domestication and Dog Domestication Vis-A-Vis

As noted, domesticated animals exhibit several distinctive features compared to their wild conspecifics. As also noted, it has been claimed that (most of) these traits are shared across (most) domesticates, to the extent that a “domestication syndrome” can be posited (Wilkins et al., 2014). These common features include body differences (floppy ears, shorter snouts, smaller jaws, smaller teeth, reduced skulls/brains, depigmentation), physiological changes (neoteny, shorter reproductive cycles, earlier sexual maturity, increased fecundity, adrenal hypofunction, reduced levels of stress hormones), behavioral differences (increased docility), and even cognitive changes (enhanced skillfulness in using human cues). According to Wilkins et al. (2014), this common set of features associated with domestication results from the impact of selection for tameness on the neural crest, an embryonic structure involved in the development of multiple body parts.

As discussed by Sánchez-Villagra et al. (2016), not all these features are present in all mammal domesticates, although dogs are a notable exception, as they show the full syndrome. Humans show many of these traits too. Accordingly, compared to extinct hominins, AMHs exhibit reduced skulls/brains (at least during the last 50 ky), reduced teeth, childish faces, neoteny, and lower levels of reactive aggressiveness (Márquez et al., 2014; Thomas, 2014; Fukase et al., 2015; Stringer, 2016; Hare, 2017), with the expression of these features of domestication having intensified recently, during the Upper Paleolithic (Cieri et al., 2014). Interestingly enough, candidate genes for mammal domestication are overrepresented among the genes under positive selection in AMHs compared to Neanderthals and Denisovans (Theofanopoulou et al., 2017). More precisely, different sets of candidate genes for animal domestication seem to have been selected at different moments during human evolution, suggesting that human self-domestication might be an ongoing process in our species (Benítez-Burraco et al., 2019). Supporting this view, features of self-domestication appear in different degrees in different human groups, depending on environmental and cultural factors. For instance, the higher status women have in society, the less sexual dimorphism one finds, as a result of a sustained preference for less-aggressive males (Gleeson and Kushnick, 2018). As noted, in mammals, domestication is usually triggered by selection for tameness. It has been hypothesized that human self-domestication also resulted from selection of less threatening and less emotionally reactive partners, as a consequence of the advent of community living (particularly when this involved extra-group individuals), co-parenting, and/or changes in human foraging ecology (Hare et al., 2012; Pisor and Surbeck, 2019). Reduced environmental stress resulting from generalized fire and tool using (Domínguez-Rodrigo and Pickering, 2017) might also have contributed to our self-domestication, considering the positive effect of decreased environmental stress conditions on prosocial behavior, as seen in bonobos (Hare et al., 2012), island wolves (Darimont et al., 2014), or Florida key deer (Harveson et al., 2007). A final factor contributing to trigger our self-domestication could be the generalized climate deterioration that occurred during the Last Glaciation (spanning from 110 kya to 15 kya), since harsh environments have been proven to favor prosocial behavior too, particularly tolerance (Spikins et al., 2021).

Regarding dog domestication, there is ample consensus that it can be traced back to the Upper Paleolithic, although it might have started as early as 40 kya in Europe and it might have occurred quite quickly (Sundman et al., 2020). Evidence is mostly paleogenetic (e.g., Thalmann et al., 2013; Botigué et al., 2017), but paleontological evidence has also been found. Hence, remains of early dogs or protodogs, some of them as older as 30 kya, have been excavated in Europe (Germonpré et al., 2009, 2012, 2015; Ovodov et al., 2011; Prassack et al., 2020). Some authors have argued that the first dogs provided no services at all, but were simply scavengers around human camps (Coppinger and Coppinger, 2001). But there is a lack of evidence for dog domestication on the waste dump (Jung and Pörtl, 2018). Even the timeline does not fit. Dogs evolved thousands of years before the first human settlements produced waste dumps. Accordingly, dog domestication was seemingly a premeditated process. About the rationales for dog domestication, humans have used dogs as a working force mostly. Even the first dogs seemingly provided general economic services, like in hunting, guarding, or traveling (Zhang et al., 2020). It is likely that dogs inherited the knowhow to hunt big mammals like mammoth from wolves (Schleidt and Shalter, 2003, 2018). Specifically, Perri (2020) describes the importance of prehistoric hunting dogs as the first animals utilized as biotechnology by humans in the Upper Paleolithic (in contrast to non-living tools, such as lithics), as well as how dogs were incorporated into existing subsistence models. Overall, this means that dogs have been living closely linked to human for several ten thousand years. Selection for social skills, mainly to improve friendliness and communication in behavior and appearance, were leading factors in dog domestication and resulted in animals being capable of working together with humans in an active form of partnership, developing complex human-analog social behaviors (Marshall-Pescini et al., 2012, 2014; Topál et al., 2019).

The first steps toward dog domestication seemingly involved individuals archaic wolves with predisposed friendly behavior (some present-day wolves are reported to maintain interspecific prosocial contacts, e.g., toward ravens, Stahler et al., 2002, or monkeys, Venkataraman et al., 2015) and AMHs starting some sort of basic interspecific prosocial communication, first in all likelihood by the need to avoid risk of injury (Hare, 2017; Kotrschal, 2018). It has been suggested that the domestication of a competitive species like the wolf was possible because humans, who are not fully adapted to a carnivorous diet, shared with incipient domesticated wolfdogs the surplus of protein of game, thus ameliorating competition between both species during the first phase of dog domestication (Lahtinen et al., 2021). These initial interactions might have also been facilitated by the fact that wolves and humans are both highly social mammals with similar family structures, including alloparenting and trusting in the prosocial care of the whole clan (Mech, 1999, 2009; Hawkes, 2003; Page et al., 2017). Similarities extend as well to their refined communication with conspecifics, including complex mimicry (Nagasawa et al., 2011) and joint attention, as well as vocal communication (Mech, 2009; Range and Virányi, 2011; Marshall-Pescini et al., 2017). Finally, both AMHs and wolves are equipped with mirror neuron mechanisms, comparable limbic functions, and a similar basic function of prefrontal inhibition (Rizzolatti and Craighero, 2004; de Waal et al., 2005; Oberman et al., 2007; Bartal et al., 2011; Kilner and Lemon, 2013; Ferrari, 2016; Welberg, 2018), enabling both species interspecific empathy and Theory of Mind (ToM), which are obviously more rudimentary in the case of wolves (Catala et al., 2017; Huber et al., 2020). All this seemingly helped start cooperation, ultimately leading to a “working together culture” (Avital and Jablonka, 2000; Wayne, 2013; Filatova et al., 2015; Foote et al., 2016; Jung and Pörtl, 2019).

Still, the two species, dogs and wolves, show remarkable behavioral differences despite their relatively recent divergence time. Since dogs and wolves are nearly identical at the level of DNA sequences, differences are expected to result mostly from dissimilar gene expression patterns in selected body regions, notably brain areas (vonHoldt et al., 2010). Accordingly, Axelsson et al. (2013) found nineteen genes important for brain function among the regions with signals of positive selection in dogs, with eight of them being involved in pathways potentially underlying behavioral changes central to dog domestication. Likewise, Cagan and Blass (2016) found signals of strong selection during the initial stages of dog domestication on multiple genes involved in the fight-and-flight response, particularly in the catecholamine synthesis pathways contributing to reduced aggression and fear toward humans. One brain region of particular interest in this sense is the hypothalamus. Whereas gene expression patterns in the hypothalamus of wolves and coyotes are very similar, seemingly because of their stable environmental conditions, in dogs a more flexible pattern is observed, plausibly due to the fast changing conditions of the human environment (Saetre et al., 2004). As noted in the previous section, the hypothalamus, as part of the HPA axis, contributes to regulating stress, aggressiveness, and sociability. Another region of interest is the hippocampus. The hippocampus of domesticated animals is larger compared to their wild conspecifics (Rehkämper et al., 2008). In tamed foxes, tameness is accompanied by global and region-specific hippocampal increases in neurogenesis due to a lower reactivity of the HPA axis (Huang et al., 2015). The hippocampus is one key component of the neural substrate supporting cognitive maps for navigating the physical space, but also the social space, including tracking social behavior and adapting to new social contexts (Montagrin et al., 2018). Domestication has proved to result in enhanced spatial learning and contextual memory (Rehkämper et al., 2008; Lewejohann et al., 2010). Interestingly too, the hippocampus plays an important role in language processing (Covington and Duff, 2016; Piai et al., 2016; Kepinska et al., 2018). According to Corballis (2019), changes in the hippocampus in the human lineage resulted in our enhanced episodic memory, that allows us to mentally travel in time, but that also supports the core feature of human language, namely, recursion. It is thus tentative to hypothesize that domestication might have contributed to enhancing communication abilities via changes in the hippocampus (more on communication in the next section), particularly because brain area is also involved in stress management (McEwen et al., 2012). Accordingly, a sound hypothesis is that the selection for tameness underlying (self-)domestication initially impacted on the role of the hippocampus in stress management, but later modified other hippocampal functions, particularly if these modifications were advantageous in the behavioral and sociocultural environment also brought about by (self-)domestication, involving prolonged and frequent contacts with other individuals and more sophisticated social practices (see Benítez-Burraco, 2021).

Finally, one interesting result of dog domestication was the rise of dog breeds, which are populations with a set of highly specified traits developed to fulfill specialized functions in human societies (Schoenebeck and Ostrander, 2014; Zhang et al., 2020). A dog breed can be clearly identified by its appearance, behavior, and genetic fingerprint. Interestingly, as recently found by MacLean et al. (2019), breed differences in behavior (including degrees of aggression, fear, trainability, attachment, and predatory chasing behaviors) are highly heritable, to the extent that clustering of breeds based on behavior accurately recapitulates their genetic relationships. Interestingly too, breed specialization is mirrored in the brain anatomy, which varies significantly between breeds, likely due to human selection for behavior (Hecht, 2019; Zapata et al., 2020). The most conspicuous alterations in the brains of different lineages of domestic dogs concern regions managing inhibitory control and communication skills toward humans (more on this on section below). For instance, as noted by Frederick (2019), brain differences in scent dogs do not concern areas involved in smelling, but instead the more sophisticated areas that help dogs understand and communicate information.

## Human–Dog Coexistence… and Co-Domestication?

It is a long way from the wolf to the first dogs, to specialized dogs, and eventually to breeds. Therefore, this would have involved thousands of years of co-living and co-working. Sled dog teams might have been used in Siberia as early as 15 kya (Pitulko and Kasparov, 2017). Genetic evidence suggests that 9.5 kya the gene flow from Siberian wolves to dogs stopped, to the extent that sled dogs from that period were very similar to modern sled dogs (Sinding et al., 2020). The oldest fossilized bones representing two clearly different types of dogs are from 9 kya: sledding and hunting dogs (Pitulko and Kasparov, 2017). Since the beginnings of the Neolithic period, there is ample evidence of dogs as specialized working partners for hunting, herding, sledding, or guarding in all continents, apart from Australia (Jung, 2011; Perri, 2016; Guagnin et al., 2018). Cave paintings and rock art from Northern Africa or the Arabian Peninsula from 9 to 10 kya show men and dogs hunting or herding together (Coulson and Campbell, 2001; Holl, 2004; Guagnin et al., 2018). As cultures and technologies developed, jobs carried out by working dogs did so as well, resulting in the separation of dogs into different types of animals selected for different working functions, and ultimately, in dozens of breeds (Jung and Pörtl, 2018). Parker et al. (2017) have proposed a two-step process of breed creation: first, separation by functional employment; later, selection for physical attributes. Overall, human social, economic, and cultural evolution is mirrored in the evolution of dog, its specializations, and its breeds. Putting this differently, we may understand dog breeds as a reflection of human cultural evolution.

To some extent, this prolonged interaction has resulted in similar physical and behavioral changes in both species. Accordingly, Tibetan dogs and Tibetan people exhibit comparable adaptive strategies for living at the high altitude of the Himalayan Mountains (Wang et al., 2014), to the extent that in both cases, positive selection signals have been attested in the same gene, namely *EPAS1* (Gou et al., 2014; Xu et al., 2014; Li et al., 2015). This can be viewed as a parallel process of adapting, just like dogs having adapted to the starch-rich diet of their human caregivers, with the copy number of genes involved in the breakdown of amylase being greater in modern companion breeds than in sledge dogs, reflecting the spread of agriculture during Prehistory (Arendt et al., 2016; Ollivier et al., 2016).

Available evidence suggests, however, that this enduring co-existence between dogs and humans cannot be reduced to an instance of a parallel evolution. As noted, humans have significantly modified dog’s body, behavior, and even cognition through active selection, this resulting in dozens of different breeds. For their part, dogs have contributed to important changes in the human society, of the sort discussed above, resulting in a “working together culture,” and eventually, in new forms of human culture, like mammoth-hunting (Shipman, 2015).

Nonetheless, we aim to push this line of reasoning further, and to regard the parallel evolution between humans and dogs as an instance of co-adaptation, or perhaps of co-evolution, and to ultimately claim that both species might have been engaged in a co-domestication process. At the beginning of dog’s domestication, hunting of large preys together with the first wolfdogs seemingly provided an increased amount of food as a resource for growing human clans, but also increased the survival rates of the former, because, as noted, humans seemingly shared with incipient domesticated wolfdogs their hunting surpluses, because of our poor adaptation to animal protein uptake (Lahtinen et al., 2021). Over time, dogs became docile and enabled humans to keep some livestock, like sheep or goats, thus contributing to establishing a farming culture, while benefiting from living in the human-made environment. This sort of link has been explicitly addressed by many as a form of dog-human co-adaption (Schleidt and Shalter, 2003, 2018; Russell, 2018; Jung and Pörtl, 2019), as already described by Darwin (1860, p. 31).

Now, our contention is that dog domestication and human self-domestication might have been involved in a positive feedback loop. In our opinion, this possibility is supported by evidence of a diverse nature, from archeological, to physiological, to genetic. We now review this evidence. In the next section we will focus on language abilities. We will argue that the enhancement of interspecific communication might not have been just a consequence of this feedback loop, but also one of its triggering factors, and that human language evolution benefited, even if subtly, from it.

First, the advent of the dog itself. As noted in the previous section, the onset of dog domestication has been claimed to be about 45–40 kya. This is the period when cumulative technological and cultural evolution of AMHs took an enormous leap into a new area of modernity (McBrearty and Brooks, 2000; Mellars, 2002), but also when features of human self-domestication reached its peak (Cieri et al., 2014).

Second, archeological findings point to dogs closely living as in-group members of human societies. Specifically, dog burials are frequent during the Paleolithic period and there are often human-dog graves, like in Oberkassel, Germany, dated 14.2 kya (Morey and Wiant, 1992; Morey, 1994; Losey et al., 2011, 2013; Janssens et al., 2018). Dog or human-dog graves have been found much more frequently than that of cats, horses, or other animals. This circumstance tells of a deep emotional link between dogs and Paleolithic people (Janssens et al., 2018; Jung and Pörtl, 2018), particularly if one considers that in the Oberkassel grave one of the buried dogs had been ill for several months and had received intensive human care before dying (Janssens et al., 2018). As noted, these intense inter-specific contacts are expected to have contributed to reducing reactive aggression in both humans and dogs and to increasing tolerance toward non-kin.

Third, interacting with dogs is known to reduce physiological signals/triggers of stress and reactive aggression in their human handlers, including heart rate, blood pressure, and pulse frequency (Friedmann et al., 1983; Grossberg and Alf, 1985; Nagengast et al., 1997; Hansen et al., 1999), cortisol level (Barker et al., 2005; Beetz et al., 2011; Kertes et al., 2017; Schretzmayer et al., 2017), and serotonin and oxytocin release (Odendaal, 2000; Odendaal and Meintjes, 2003; Miller et al., 2009; Nagasawa et al., 2009a,b; Handlin et al., 2011; Beetz et al., 2012). Cortisol is known to control the fight-and-flight response and, ultimately, reactive aggression (van Bokhoven et al., 2005; Böhnke et al., 2010; Montoya et al., 2012). Reduced levels of cortisol and other stress hormones resulting from reduced sensitivity of the HPA axis after selecting for tameness have been claimed to contribute to the behavioral changes associated with domestication (Künzl and Sachser, 1999; Trut et al., 2009). Regarding oxytocin, this is one neuropeptide providing in-group bonding and out-group rejection (Kosfeld et al., 2005; Zhang et al., 2019). In humans, this is one of the hormones subject to sexual selection with respect to a reduction in physically aggressive behavior in the context of our self-domestication (Hare, 2017).

Fourth, this close relationship between dogs and humans can impact on key cognitive abilities via brain changes promoted by the physiological mechanisms involved in in-group affiliation and stress management. As we discuss in the following section, many of these abilities are needed for proper language acquisition and processing. Accordingly, decreased cortisol levels have been shown to cause dendritic growth (McEwen and Morrison, 2013), ultimately improving, neural structures, that are crucially involved in social learning and inhibitory control, like the prefrontal cortex (Arai et al., 2009; Brusini et al., 2018). Compared to wolves, dogs possess a higher level of inhibitory control towards humans, as measured using the ‘cylinder task,’ with dogs showing a less aggressive behavior even toward foreign people (Marshall-Pescini et al., 2015). Likewise, higher levels of oxytocin, when delivered intranasally, result in dogs having an enhanced ability to perform an object choice task involving the use of human pointing cues (Oliva et al., 2019). Oxytocin also enhances joint attention (Nagasawa et al., 2015) as a link to object learning (Marshall-Pescini et al., 2012, 2014). As noted by Meaney and Szyf (2005), reduced stress levels and enhanced prosocial behavior can modulate brain function epigenetically. Accordingly, as also found among humans, in rats, maternal behavior toward offspring provokes long-term changes in the response to stress that result from changes in gene expression impacting on the HPA axis. In our view, reduced stress activity within a mixed human-wolves clan can be hypothesized to have had an evolutionary benefit also at the cognitive level. Thus, from generation to generation, cortisol sensitivity might have decreased more and more, while the cross regulated sensitivity for prosocial neurotransmitters and neuropeptides like serotonin and oxytocin might have increased steadily, eventually engaging in a positive feedback loop.

Fifth, in dogs and humans, there are common genetic signatures of selection in physiological processes involved in domestication. In particular, mutations and changes in methylation patterns in the oxytocin receptor gene, *OXTR*, have been found in samples of dogs compared to wolves, providing evidence that this gene might have played an active role in dog domestication (Oliva et al., 2016; Shilton et al., 2020; vonHoldt et al., 2020). Likewise, signatures of positive selection in *cis*-regulatory sequences of *OXTR* have been found in humans (Schaschl et al., 2015). And in both humans and dogs the genetic variation of *OXTR* is associated to differences in social behavior (Eales, 1989; Pfenning et al., 2014; Shilton et al., 2020). By contrast, this correlation is not found in wolves (Nagasawa et al., 2015).

Sixth, more generally, there is a convergence between the genetic bases of dog domestication and human self-domestication. Accordingly, genes positively selected in humans compared to extinct hominins are enriched in candidates for mammal domestication, particularly dog domestication (Theofanopoulou et al., 2017). Intriguingly, this overlapping also encompasses highly prevalent human cognitive diseases impacting on social and communicative abilities, which present with altered features of self-domestication. For instance, genes that are found dysregulated in the blood of people with autism spectrum disorder (ASD) are enriched in candidates for mammal domestication (Benítez-Burraco, 2020). People with ASD show attenuated features of human self-domestication (Benítez-Burraco et al., 2016). Likewise, a genomic region found to be under positive selection in domestic dog breeds causes WS in humans when deleted (vonHoldt et al., 2017). WS is a clinical condition resulting from the loss of around 30 genes from one of the copies of chromosome 7 (Pober, 2003). Subjects with this condition exhibit a distinctive phenotype, including altered growth patterns, craniofacial anomalies, heart disease, intellectual disability and impaired visuospatial cognition, spared sociability and musical abilities, and notable language abilities (Mervis and Becerra, 2007; Morris, 2010; Pober, 2010). Interestingly, WS also involves exaggerated features of human self-domestication (Niego and Benítez-Burraco, 2019). Furthermore, genes dysregulated in the blood on people with WS are enriched in candidates for animal domestication (Niego and Benítez-Burraco, 2019). At the same time, hypersociability, a central feature of dogs compared to wolves, can be linked to structural variants of *GTF2I* and *GTF2IRD1*, two of the genes within the WS region (vonHoldt et al., 2017), but also to the altered expression of several other genes located in this region, including *BAZ1B* (vonHoldt et al., 2018). *BAZ1B* is a robust candidate for domestication in mammals (Wilkins et al., 2014). It also contributes to regulating the balance between neural precursor self-renewal and differentiation (Lalli et al., 2016). Zanella et al. (2019) found a modern-specific enrichment for regulatory changes both in the human *BAZ1B* and its downstream targets. Finally, in people with WS, the *OXTR* gene is found hypomethylated, seemingly as a result of the altered functioning of methyltransferase genes located within the WS locus (Haas and Smith, 2015). Thus, for bith humans and dogs, an interface between selected genes within the WS region and the (epigenetic modulation of the) HPA axis can be hypothesized, with this interface impacting on the serotonin and oxytocin systems, and ultimately, on social behavior. Overall, these molecular findings suggest that in dogs, selection toward domestication targeted a limited set of behavioral genes with larger phenotypic effects that facilitated the coexistence with humans, with several of them being located within the WS region. But the same might have occurred in humans, with alterations of genes within the WS region playing a key role in the evolution of the modern human face and prosociality.

Sixth, human–dog contacts might rescue abnormal self-domestication features in humans. As noted, diverse clinical conditions entailing behavioral, cognitive, and language problems, like ASD and WS, also exhibit altered features of human self-domestication. Studies have shown positive effects of dog facilitated therapy on people with ASD, including increased social interaction, enhanced social learning abilities and communication, and reduced stress (Sams et al., 2006; Prothmann et al., 2009; Beetz et al., 2012; O’Haire, 2013; Julius et al., 2014; Carlisle, 2015; Siewertsen et al., 2015; Wijker et al., 2019). Noticeably, people on the ASD spectrum are often more interested in social and communicative contact with dogs than with humans (Siewertsen et al., 2015; Valiyamattam et al., 2019). Likewise, people with schizophrenia (SZ), who exhibit exaggerated signs of self-domestication at the physical, behavioral, and even brain/cognitive levels (Benítez-Burraco et al., 2017), benefit from dog facilitated therapy, showing more prosocial interactions with other patients (Nathans-Barel et al., 2005; Villalta-Gil et al., 2009). In turn, these effects of dog facilitated therapy can be linked to specific epigenetic and physiological aspects of stress response and prosocial behavior involved in human-dog attachment, and eventually, in human–dog co-domestication, as described above. To put just one example, ASD is characterized by abnormally low levels of oxytocin (Modahl et al., 1998), reduced density of OTXR in the brain (Haas and Smith, 2015), and the hypermethylation of the promotor region of *OXTR* (Haas and Smith, 2015; Andari et al., 2020). These molecular features correlate with clinical symptoms and with a reduced connectivity between cortico-cortical areas involved in ToM (Andari et al., 2020). Increased levels of oxytocin via nasal delivery result in increased attention to social stimuli and their understanding by subjects with ASD (Domes et al., 2013; Gordon et al., 2016). It could be thus hypothesized that interactions with dogs might increase oxytocin levels and ultimately result in the improvement of patients’ social, emotional, and cognitive functioning. Although there is still no direct evidence for this effect, an analogy can be drawn with oxytocin increases due to dog–human interactions in neurotypical groups for which there is ample evidence (Odendaal, 2000; Odendaal and Meintjes, 2003; Miller et al., 2009; Nagasawa et al., 2009a,b; Handlin et al., 2011; Beetz et al., 2012; Wijker et al., 2019; Powell et al., 2020). Eventually, this effect could have a direct impact on communicative (dis)abilities: as noted by Nagasawa et al. (2015), gazing behavior from dogs, which is crucially involved in human-like modes of communication, increases urinary oxytocin concentrations in owners, this favoring affiliative behavior, which in turn results in increased oxytocin concentration in dogs. Supporting this human–dog oxytocin mediated positive loop modulated by gazing, it is interesting to note that nasally administered oxytocin increases gazing behavior in dogs, also resulting in increased oxytocin concentrations in owners. Likewise, reduced cortisol awakening responses compared to their peers have been observed in children with ASD with contact to an assistance dog (Viau et al., 2010).

Finally, the close bonding between humans and dogs, closer in fact than between any other species, results in a high number of diseases suffered by both, especially cognitive disorders, which reflect a similar lifestyle with intimate contacts between species (Zhang et al., 2020). Interestingly, some of these diseases can be related to (self-)domestication, particularly, conditions affecting cognition and behavior. On the human side, as noted, highly prevalent diseases impacting on our cognitive architecture and our distinctive behavior, like ASD, SZ, or WS, have been linked to an abnormal presentation of self-domestication features. On the dog side, many diseases can be construed as cognitive disorders too (Shearin and Ostrander, 2010; Ostrander et al., 2017). In some cases, we do find common etiological factors. One interesting instance is canine compulsive disorder (CCD), which parallels human obsessive compulsive disorder (OCD). Both CCD and OCD have been associated with the *CDH2* gene (Dodman et al., 2010; Moya et al., 2013; Tang et al., 2014). Variants of *CDH2* can also contribute to Tourette syndrome (TS), a tic disorder which is sometimes accompanied by production of derogatory language (Moya et al., 2013). CCD, OCD, and TS are hereditary conditions that seem to be triggered by environmental stressors and result in abnormal patterns of inhibition/disinhibition (mostly imbalanced serotonergic and dopaminergic pathways) in the cortico-striatal-thalamic-cortical loop (Vermeire et al., 2012). This loop is involved both in the inhibition/suppression of aggression and in syntactic/vocal signal chunking (see Benítez-Burraco and Progovac, 2021 for details). TS has been highlighted as a proxy or window to previous stages in human self-domestication entailing higher levels of reactive aggression (see Progovac and Benítez-Burraco, 2019 for details).

## A Rationale for Human–Dog Co-Domestication

At the core of the suggested feedback loop between dog domestication and human self-domestication, we find the increased prosocial behavior and the more complex social and cultural practices brought about by our increased self-domestication as the factor stimulating the specialization of dogs, this in turn resulting in different breeds selected for different functions (Jung and Pörtl, 2019). At the same time, we find dog domestication as an active factor promoting human self-domestication, as increased interspecific contacts are expected to have reinforced our trend toward enhanced prosociality (Jung and Pörtl, 2015, 2018, 2019). Current research shows that human-dog relationships provoke similar patterns of brain activation in areas involved in reward, emotion, affiliation, and empathy than human-infant interactions, although some differences can be observed as well (see Vanutelli and Balconi, 2015 for review). Interestingly, as noted, domestication results in neotenic, childish features, which are expected to increase our affiliative and empathic responses, and ultimately, to promote our prosociality. For instance, research conducted by Archer and Monton (2010) showed that infant features in the faces of dogs (young or adult) function as social releasers and evoke an affiliative response in humans, with pet owners who are more strongly attached to their pets showing stronger preferences for images of animals with infant features. According to our view, the ultimate triggering factor of the hypothesized feedback loop would have been the integration of the wolf into the human social structure, which was previously characterized by family-based groups with only little permeability, with the only exception of the assimilation of mating partners to avoid inbreeding (Sikora et al., 2017). Elsewhere, we have called this view the hypothesis of the “active social domestication” (Jung and Pörtl, 2015; Pörtl and Jung, 2017, 2019).

According to our view, this cohabitation and communication with wolves (and later, with dogs) did not only resulted in reduced reactive aggression (to avoid conflicts with a non-kin species), thus potentiating self-domestication features as described above, but also, as time went by, in a potentiation of selected cognitive aspects, many of them important for language acquisition and use, particularly ToM (to avoid misunderstandings and miscommunications with a non-kin species). ToM comprisses a verbal ToM and a non-verbal ToM, and both rely on different brain systems (Wiesmann et al., 2020). Specifically, non-verbal ToM relies on the gyrus supramarginalis, which is involved in emotional, social, and visual attention. Our view is that interspecific contacts with wolves/dogs enhanced non-verbal ToM firstly, thus strengthening emotional and social attention. Later, other cognitive capabilities might have been reinforced, including verbal ToM (more on this below).

Finally, regarding a potential neurobiological mechanism accounting for these coordinated changes, we contend that it might have involved a set of cordinated changes in the interactions between the HPA axis and the 5-Hydroxytryptamine (5-HT) system. Hippocampal glucocorticoid receptor density (hGCR) has an inhibitory effect on this system. Prosocial behavior promotes an increase in serotonin levels which upregulates hGCR via selected epigenetic changes, this also resulting in decreased cortisol levels. In turn, low cortisol levels increase social learning capabilities and promote the activity of the prefrontal cortex, contributing to improving executive functions like cognitive inhibition, ToM, working memory, and language skills (see Pörtl and Jung, 2017, 2019 for details). A second mechanism of interest concerns the dopamine reward system, which exhibits differences in domesticated animals compared to their wild conspecifics (Komiyama et al., 2014; Sato et al., 2020). Changes in dopamine signaling might have thus contributed not only to more diverse and complex vocal signals in both dogs and humans as noted in the Introduction, but also to potentiating dog-human co-adaptation, and perhaps co-evolution. One reason is the positive feedback loop between language complexity and human self-domestication features, which ultimately entails that complex communication could promote features of domestication (see Progovac and Benítez-Burraco, 2019 for details).

## Language Evolution in a Potential Scenario of Human–Dog Co-Evolution

In the last part of the paper, we will hypothesize about the consequences of a putative human-dog co-domestication scenario on the evolution of communication abilities. On the dog side, domestication seemingly involved the emergence (or the reinforcement) in first wolfdogs of human-like social skills important for communicating, particularly, joint attention (Nagasawa et al., 2015), the following of (human) referential gestures (Kaminski and Nitzschner, 2013; Range and Virányi, 2013), and over-imitation, that is, the eagerness to copy causally irrelevant actions, which is prevalent in humans, but absent in great apes (Huber et al., 2020). Contrary to socialized wolves, dogs look back at humans when confronted with an insoluble problem: this attentiveness to the human face suggests that dog-human communication is more complex and cannot be achieved by wolves even after extended socialization (Miklósi et al., 2003). Also, dogs have not only learned to interpret human gestures, including cues for help, but to look for them actively, very similarly to how human toddlers do (MacLean et al., 2017). Even free-ranging dogs can understand human cues, including complex human pointing gestures (Bhattacharjee et al., 2020). Dogs are capable of recognizing the finest human emotions (Albuquerque et al., 2016). At the same time, dogs play an active role in this interspecific non-verbal communication. For instance, getting human attention increases the number of facial movements and expressions by dogs, this being suggestive of some communicative intention (Kaminski et al., 2017). Facial movements addressed to humans differ from facial expressions directed to their conspecifics (Caeiro et al., 2017). Evolutionarily, we can trace back these changes to modifications in the facial muscle anatomy (Diogo et al., 2018; Sexton, 2019). Accordingly, in the very short time period of the evolution from wolves to dogs, a completely new muscle was a added, the levator anguli oculi medialis, which is responsible for raising the inner eyebrow intensely and which supports facial movements that are interpreted by human caregivers as a social bonding cue, e.g., as a friendly begging (Kaminski et al., 2019; Sexton, 2019). In this sense, Salomons et al. (2021) tested the cooperative communication abilities of dog and wolf puppies with humans. Their results support a role of domestication in enhancing the cooperative communication skills of dogs with human, involving changes in different developmental pathways. As a matter of fact, also our distinctive face, which is short and retracted beneath a large globular braincase compared to other hominins, has been hypothesized to have evolved under the effect of social influences (Lacruz et al., 2019). Specifically, Sánchez-Villagra and van Schaik (2019) have suggested that it might have acted during our evolution as a signal of friendliness and social tolerance, contributing to increased in-group and, particularly, inter-group contacts. These childish features of the human face can be linked to our self-domestication, as domestication commonly results in neotenic features (see Wilkins et al., 2014 for discussion).

Nonetheless, it is not only non-verbal communication abilities that have improved in dogs as a result of contact with humans. Dogs seem to have acquired a special ability for interpreting human oral language (Fugazza and Miklósi, 2020). Border collies are able to understand the names for more than 1,000 objects (Pilley and Reid, 2011). Moreover, dogs are able to identify new objects only by their names (Fugazza and Miklósi, 2020), which involves a notable capability for fast mapping, i.e., inferring the names of novel items by exclusion (Kaminski et al., 2004). fMRI experiments suggest that words and non-words are processed in dogs by different neural networks, and relying on different basic processes like novelty detection, as well as auditory and hedonic representations (Prichard et al., 2018). Interestingly, similarly to humans, dogs also process words with a hemispheric bias, with lexical and intentional aspects being processed separately (Andics et al., 2016). These skills are remarkable if one considers that dog barking lacks most of the features defining human language, particularly, compositionality and duality of patterning. During dog domestication, barking became the prevalent mode of vocal communication for dogs, e.g., as a hunting partner (Perri, 2020). Wolves only bark very briefly and only in rare cases (Mech and Boitani, 2010; Faragó et al., 2014). Dog barking can thus be interpreted as an adaptation to human speech-based communication (Pongrácz et al., 2010; Paladini, 2020). In this sense, it is intriguing that several genes within the WS region have been positively selected in dogs, as noted in the previous section, if one considers that subjects with WS show enhanced musicality and expressivity, commonly manifested through a heightened emotional responsiveness to music (Thakur et al., 2018).

On the human side, as also discussed in the previous section, our self-domestication resulted in reduced levels reactive aggression, hypersocial behavior, and increased cooperation skills. In turn, these changes seemingly brought about improved social learning, abilities, an enhanced working memory, greater emotional inhibition, better executive functions, and a improved ToM, with these modifications ultimately promoting changes in brain function and anatomy due to the increased interspecific prosocial contacts (see Pörtl and Jung, 2017; Jung and Pörtl, 2018 for details). All these are important changes for achieving enhanced communication abilities. In particular, as noted in the Introduction, they are expected to have contributed to those aspects of languages that are thought to result from a cultural process, specifically to the structural complexity of modern languages (Thomas and Kirby, 2018; Benítez-Burraco and Progovac, 2020), with increasingly sophisticated verbal behavior and enhanced self-domestication features being involved in a positive feed-back loop (Progovac and Benítez-Burraco, 2019). Modern uses of languages (i.e., pragmatics) are also expected to have been (re)modeled by our increased self-domestication, particularly because reduced reactive aggression and enhanced prosocial behavior seemingly facilitated the potentiation of pragmatic principles governing conversation, including turn-taking and conversational implicature see Benítez-Burraco et al. (2020) and references herein for further discussion. If man-dog coexistence and interaction contributed to potentiating our self-domestication features, as argued in the previous section, they should have had some impact as well on our language abilities, including language structure and use. There are not many studies examining in detail how human communication patterns might be affected by (changes in) dog communication. Interestingly, there seems to exist a “dog-directed speech,” which is the type of “language” addressed to dogs that parallels the “child-directed speech” or “motherese” which plays an important role in language acquisition by the child. Both puppies and adult dogs seem to attend to, and show more affiliative behavior toward people using dog-directed speech, with this effect resulting from a combination of the acoustic properties and the contents of the specific words encompassing this type of speech (Benjamin and Slocombe, 2018). It also happens that bonding with dogs also results in increased verbal communication attempts toward other non-human animals (Epley et al., 2008). Finally, interacting with dogs has been claimed to have a positive impact on reading skills (Hall et al., 2016). But admittedly, these are circumstantial findings and more research on this issue is needed.

Overall, selection for tameness and cooperation, and therefore, for less aggressive phenotypes and increased self-control, can be expected to have resulted in changes in verbal and non-verbal communication in both dogs and humans. Ultimately, it is tentative to hypothesize that AMHs improved, even subtly, their ability to communicate with non-kin groups by first interacting with non-kin species (wolf/dog). After all, dog domestication predates the emergence of extensive human social networks involving non-related individuals, which become generalized only during the Magdalenian period, around 12 kya (Vanhaeren and d’Errico, 2006; Schwendler, 2012). This ability for interacting with non-kin underlies the last stage in the evolution of human languages, to be precise, the emergence of the so-called *exoteric* languages, that are better suited for conveying decontextualized meanings to strangers (see Benítez-Burraco and Progovac, 2020 for details).

## Conclusion

In this paper, we have explored the consequences of human-dog interactions for the evolution of their respective cognitive and behavioral distinctive features, with a focus on communication. Our main interest has been the parallels between dog domestication and human self-domestication. We have hypothesized that this parallel evolution might have involved some sort of co-evolution, mostly through an impact on selected mechanisms controlling reactive aggression and prosocial behavior. On the dog side, this would have resulted in an increased sociability toward humans and an increased sensibility and responsiveness to human communicative signals. On the human side, this would have contributed to increasing our trend toward a self-domesticated phenotype, ultimately favoring the emergence of more complex forms of language through a cultural mechanism.

**FIGURE 1:**
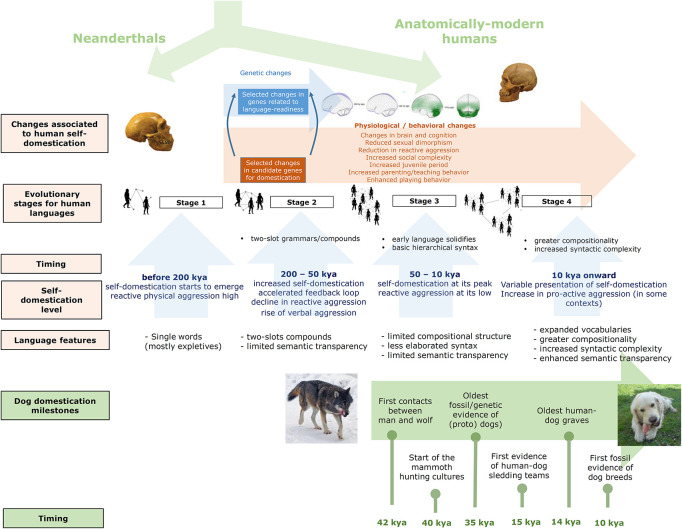
A graphical summary of our model of language evolution under the forces of self-domestication and the hypothesized feedback effect with dog domestication. The images of the wolf and the dog are from Wikipedia (CC BY-SA 3.0).

Needless to say, the hypothesized effect of human–dog interactions on the evolution of present-day languages can be expected to be quite subtle. Language evolution and language change certainly result from the interplay of multiple factors. Above all, our faculty for learning and using languages seemingly resulted from some human-specific brain changes that habilitated a new neuronal workspace that brought about our characteristic ability for conceptual merging and our distinctive cognitive fluidity. At the same time, most if not all cognitive components supporting this ability (and language, more generally) exhibit a notable evolutionary continuity, with precursors in other species. Behavioral changes were also crucial in the path toward modern languages, but as with the cognitive pieces of language, behavioral aspects also exhibit a long evolutionary history. Being an important factor, self-domestication by itself cannot be regarded as the sole force accounting for the emergence of present-day languages, as other species having experienced domestication and self-domestication, like bonobos, have not developed a complex language. Accordingly, a more nuanced view is that self-domestication favored the creation of the suitable environment (mostly behavioral, but perhaps cognitive too) that enabled us to put to use our human-specific innovations (mostly cognitive, but perhaps behavioral too). In this context, our prolonged interaction with dogs would have been one among many factors contributing to reducing our reactive aggression and to stimulating our self-domestication, thus standing out as one minor piece of a bigger puzzle. Our whole hypothesis is summarized in Figure 1.

Still, we think that our hypothesis is based on solid evidence and set forth several testable predictions that open new avenues for future research. For instance, in the domain of genetics, it could be interesting to delve into the analysis of the convergent signals of domestication found in humans and dogs, looking for a rationale for the observed behavioral and cognitive changes, but also for evidence of the hypothesized co-evolution through a gene-culture mechanism. Likewise, it would be interesting to know more about patterns of interactions, with a focus on communication abilities, between dogs and people suffering from conditions entailing altered socialization patterns/aggression management and abnormal features of self-domestication, particularly ASD. Finally, as dogs seem to have been fully domesticated in Eurasia, it could be interesting to look for differences in the aspects highlighted in the paper between human groups from this region and peoples from places where dogs arrived much later, like South Africa or Australia.

## Data Availability Statement

The original contributions presented in the study are included in the article, further inquiries can be directed to the corresponding author.

## Ethics Statement

Ethical review and approval was not required for the study on human participants in accordance with the local legislation and institutional requirements. Written informed consent for participation was not required for this study in accordance with the national legislation and the institutional requirements. Ethical review and approval was not required for the animal study because our study is based on previous published research. Written informed consent for participation was not obtained from the owners because our study is based on previous published research.

## Author Contributions

AB-B, DP, and CJ conceived and wrote the manuscript. All authors contributed to the article and approved the submitted version.

## Conflict of Interest

The authors declare that the research was conducted in the absence of any commercial or financial relationships that could be construed as a potential conflict of interest.

## Publisher’s Note

All claims expressed in this article are solely those of the authors and do not necessarily represent those of their affiliated organizations, or those of the publisher, the editors and the reviewers. Any product that may be evaluated in this article, or claim that may be made by its manufacturer, is not guaranteed or endorsed by the publisher.
